# Retrospective analysis of tricuspid valve repair using a novel surgical technique: A 7-year single-surgeon experience

**DOI:** 10.1177/0267659120910373

**Published:** 2020-04-27

**Authors:** Ashiq Abdul Khader, Habib Khan, Catherine Stowell, Guiqing Liu, Mohamed Sameh, Prakash Punjabi

**Affiliations:** 1 Department of Medicine, Faculty of Medicine, Imperial College London, London, UK; 2 Cardiothoracic Surgery, Department of Surgery and Cancer, Imperial College London, London, UK; 3 Department of Echocardiography, Imperial College Healthcare NHS Trust, Hammersmith Hospital, London, UK; 4 Imperial College Faculty of Medicine, National Heart and Lung Institute, Hammersmith Hospital, London, UK

**Keywords:** tricuspid valve surgery, tricuspid repair, novel suture annuloplasty, cardiac surgery, tricuspid valve

## Abstract

**Objectives:**

Tricuspid annuloplasty is the optimal surgical repair technique for tricuspid regurgitation which improves mortality and morbidity. Ring annuloplasties is the techniques of choice. Here, we evaluate the efficacy and durability of a new method of interrupted pledgeted suture annuloplasty.

**Methods:**

Between 2011 and 2018, 39 eligible patients underwent tricuspid valve repair using this novel technique. Indication for repair was a grade of regurgitation at moderate or greater, or an annular diameter >40 mm. Patients were assessed both preoperatively and postoperatively by echocardiogram. Follow-up results were split into the first postoperative echocardiogram and most recent postoperative echocardiogram undertaken.

**Results:**

There were two in-hospital mortalities and two patients required permanent pacemaker implantation following surgery. At the time of the first postoperative echocardiogram undertaken (median 3 months postoperatively), freedom from moderate-severe regurgitation was 92.3%. At the time of the most recent postoperative echocardiogram undertaken (median 11 months postoperatively); none or mild regurgitation was detected in 24 patients (61.5%), mild-moderate in 11 (28.2%) and moderate-severe in 4 (10.3%) patients. Freedom from moderate-severe regurgitation was 89.7%. Postoperative grade of regurgitation was significantly reduced from preoperative grades (p < 0.001).

**Conclusion:**

Initial and midterm results of our technique show a good durability of repair. We have demonstrated recurrence rates of regurgitation equal and superior to current forms of suture annuloplasty published in the literature. This novel method of suture annuloplasty can be considered in the surgical repertoire of tricuspid valve repair techniques.

## Introduction

In the context of valvular heart disease, the tricuspid valve (TV) has traditionally been largely ‘forgotten’. This is particularly pertinent with regards to cardiac surgical intervention for TV disease – in the past, it was thought that functional regurgitant tricuspid disease would resolve with mitral valve surgery, rendering conservative management for tricuspid regurgitation (TR) sufficient.^1^ However, in recent times, evidence has suggested that this is not the case; represented by a large increase in annual rates of tricuspid intervention.^2^ As the field has changed to a more liberal approach to surgical treatment for TV disease, the scope for different surgical techniques has broadened. There is still debate over which type of surgery is preferable,^3^ particularly in terms of method of valve repair. Hence outcome reports of novel repair techniques provide value to the surgical repertoire of TV repair. Here, we report a new method of repair, using interrupted pledgeted sutures.

Severe TR has been shown to decrease survival (63.9% 1-year survival rate in patients with severe TR compared with a 91.7% 1-year survival rate in those without TR) independent of left-sided heart disease.^4^

Current surgical approaches to TV repair mainly consist of either suture or ring annuloplasties.^5^ Ring annuloplasty utilizes rigid or semi-rigid rings to fix the annulus, aiming to restore normal geometry of the valve. The ring is sized, for example, by measuring the size of the septal annulus, as it dilates the least in functional TR.^6,7^ While there is no complete consensus of which technique is superior, surgeon preference plays a key role in the decision-making process of approach to TV repair. Here we report a novel technique of suture annuloplasty for TV repair to treat functional TR, to help add to the options available to cardiac surgeons. An analysis of our experience of this procedure and its effectiveness in reducing grade of TR forms the basis of this article.

## Methods

Between May 2011 and December 2018, 43 patients underwent TV repair suture annuloplasty using an interrupted pledgeted technique. Indications for TV repair were a grade of regurgitation ⩾moderate, or an annular diameter of >40 mm in patients with less than moderate regurgitation. The aetiology of TR was secondary, due to annular dilatation, in all patients. All patients underwent concomitant cardiac procedures at the time of TV repair; preoperative and surgical characteristics (Table 1) highlight mitral valve repair/replacement as the most common concomitant procedure. Other concomitant procedures include, aortic valve replacement, coronary artery bypass grafting and atrial surgery (removal of right or left atrial appendages or closure of atrial septal defect). Of note preoperatively, three patients (7%) had mild TR, 25 (58.1%) with mild-moderate TR, and 15 (34.9%) with moderate-severe TR. Functional status of these patients with regards to comorbidities are shown in Table 2.

**Table 1.** Patient preoperative and surgical characteristics.

Characteristic	No.	%
Age (mean years ± SD)	63 ± 15	
Gender (M/F)	15/28	35/65
Preoperative TR grade		
None	0	0
Mild	3	7.0
Mild-moderate	25	58.1
Moderate-severe	15	34.9
Preoperative MR grade		
None	2	4.7
Mild	8	18.6
Mild-moderate	6	14.0
Moderate-severe	27	62.8
Preoperative RV dysfunction	14	32.6
Preoperative LVEF (mean %, ±SD)	55.2 ± 9.2	
Bypass time (mean minutes, ±SD)	130.4 ± 42.4	
Cross-clamp time (mean minutes, ±SD)	97.6 ± 32.0	
Preoperative PASP (mean mmHg ± SD)	56.6 ± 20.1	
Concomitant procedures		
MV surgery	39	90.7
AVR	10	23.3
CABG	4	9.3
AF ablation	9	20.9
Atrial surgery	13	30.2
Pacemaker implantation	1	2.3

AF: atrial fibrillation; AVR: aortic valve replacement; CABG: coronary artery bypass grafting; LVEF: left ventricular ejection fraction; MR: mitral regurgitation; MV: mitral valve; PASP: pulmonary artery systolic pressure; RV: right ventricle; TR: tricuspid regurgitation.

Total number of patients, N = 43.

**Table 2.** Patient comorbidities.

Comorbidity	No.	%
MV disease	42	97.7
AV disease	22	51.2
Preoperative angina status		
None	24	55.8
Slight limitation of ordinary physical activity	3	7.0
Symptoms at rest	7	16.3
Marked limitation of ordinary physical activity	9	20.9
Preoperative dyspnoea status		
None	2	4.7
Slight limitation of ordinary physical activity	10	23.3
Symptoms at rest	29	67.4
Marked limitation of ordinary physical activity	2	4.7
History of pulmonary disease	4	9.3
History of neurological disease		
TIA	1	2.3
CVA with full recovery	2	4.55
CVA with residual deficits	1	2.3
AF	24	55.8

AF: atrial fibrillation; AV: aortic valve; CVA: cerebrovascular accident; MV: mitral valve; TIA: transient ischemic attack.

Total number of patients, N = 43.

Patients whose preoperative or postoperative echocardiograms were not undertaken/not available for retrieval (either due to mortality or loss of follow-up) were excluded from analysis, leaving 39 patients.

### Echocardiographic assessment

Patients underwent preoperative and postoperative echocardiographic assessment of valve function either via transthoracic or transoesophageal echocardiography. The degree of TR was evaluated qualitatively by Doppler colour flow imaging and graded from no TR to severe TR. All echocardiogram reports were evaluated fully and parameters for; left ventricle (LV) function, right ventricle (RV) function, right atrial size and pressure, mitral valve regurgitation, tricuspid annular plane systolic excursion (TAPSE) and pulmonary artery systolic pressure (PASP) were recorded whenever they were present, in a total of 125 echocardiograms in these patients. In order to utilize as much echocardiogram data as possible, TV repair durability using grade of regurgitation was analysed using the first echocardiogram performed post operation and the most recent echocardiogram performed in hospital or cardiac clinic, to showcase follow-up performance.

### Operative technique

**Figure 1.**
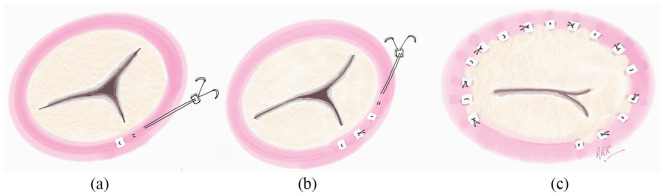
Novel method of interrupted suture annuloplasty: (a) The first pledget is embedded at the posteroseptal commissure, exit pledgets emerge 6-8 mm from the entry pledget. (b) The first suture is tied and cut; the interrupted pattern is repeated along the circumference of the annulus. (c) The final repair consists on average of eight sutures, which are each double pledgeted. The last suture is placed at the anteroseptal commissure.

Following all other concomitant procedures, the TV was exposed via an oblique incision. Our novel technique of suture annuloplasty involved reduction in tricuspid annular dimension using interrupted pledgeted sutures (Figure 1).^7^

Sutures were embedded into the annulus starting at the posteroseptal commissure. Two 2-0 Ethibond sutures within a pledget were passed into the annulus, with an exit pledget emerging 6-8 mm from the first pledget, before being tightened, tied down and cut. This interrupted suture pattern was repeated circumferentially along the annulus up to the anteroseptal commissure, ensuring avoidance of the conduction bundle. On average, eight sutures were embedded along the annular circumference, and each suture was double pledgeted. An on-table water test was performed to test competency of the valve, and if greater competency was required, additional sutures were implanted crossing the posteroseptal commissure into the septal leaflet annulus.

### Statistical analysis

Paired ordinal data were statistically compared using a Wilcoxon Signed-Rank test. A p value < 0.05 was deemed statistically significant throughout. These analyses were computed using SPSS Statistics, version 25.0 (Armonk, NY: IBM Corp). Graphs were computed using the programme Prism (Graph Pad Inc, San Diego, CA).

## Results

### Initial results

Of the original population of 43 patients undergoing TV repair, there were two surgical mortalities (4.7%) within 30 days of operation. These deaths were as a result of multi-organ failure. Preoperatively these patients had severe functional angina and dyspnoea (two patients), concomitant atrial fibrillation (AF) (one patient), and diastolic LV failure with impaired RV function (one patient). Two patients (4.7%) required permanent pacemaker implantation following surgery, one due to atypical flutter and another due to complete heart block.

Considering the 39 patients with available preoperative and postoperative echocardiogram data, the first postoperative echocardiograms (median 3 months postoperatively ranging from 3 days to 38 months) demonstrated no TR in 5 patients (12.8%), mild TR in 22 patients (56.4%), mild-moderate TR in 9 patients (23.1%) and moderate-severe TR in 3 patients (7.7%). In patients with severe TR, there was severe TR present preoperatively, with one patient re-presenting with another case of infective endocarditis of the valve 6 months after surgery.

At this time, with a median postoperative follow-up of 3 months, freedom from moderate-severe TR was 92.3%.

### Longer term: durability of TV repair

There were three mortalities during the follow-up period after discharge from hospital. In one patient, the cause of death was unable to be retrieved. In the remaining two patients, one died 11 months postoperatively due to complications from pneumonia and the other died 6 months postoperatively due to a new case of infected endocarditis. Within the follow-up period, no patients underwent redo TV surgery. Follow-up to assess for recurrence of TR in the clinic at any time after discharge from hospital was complete in 97% of patients eligible for analysis.

**Figure 2.**
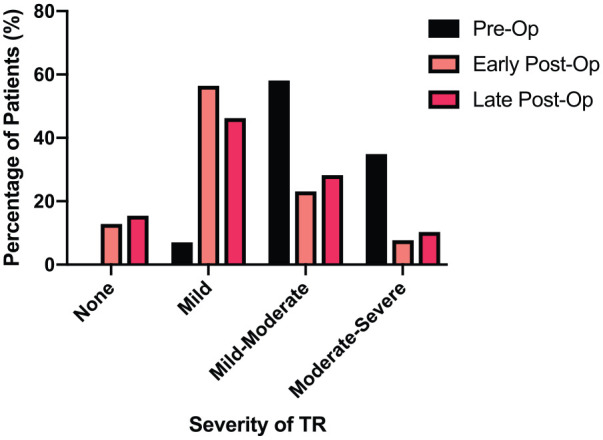
Change in prevalence within each grade of tricuspid regurgitation between preoperative and postoperative status. N = 39 both preoperatively and postoperatively.

In these 39 patients, the postoperative echocardiograms (median 11 months postoperatively, ranging from 8 days to 73 months) demonstrated no TR in 6 patients (15.4%), mild TR in 18 patients (46.2%), mild-moderate TR in 11 patients (28.2%) and moderate-severe TR in 4 patients (10.3%). Figure 2 shows the change in the prevalence of regurgitation in each of the grades between preoperative and postoperative findings. Of the four patients who were found to have moderate-severe TR during follow-up; two patients are asymptomatic but actively being monitored with a view to surgery if required, one patient was deemed too high risk for redo due to extensive comorbidities, and another patient was a subsequent mortality due to a new case of infective endocarditis.

Grades of TR were nominally ranked on a scale: none = 0, mild = 1, mild-moderate = 2, moderate = 3, moderate-severe = 4 and severe = 5; these ranks were then used in a Wilcoxon Signed-Rank test to evaluate changes in the grades of TR following TV repair. This indicated that postoperative TR grade (mean rank = 1.69) was significantly reduced compared to preoperative TR grade (mean rank = 3.31), Z = –4.7, p < 0.001. At this time, with a median postoperative follow-up of 11 months, freedom from moderate-severe TR was 89.7%. The prevalence of regurgitation at all three time points is shown in Table 3.

**Table 3.** Preoperative and postoperative tricuspid regurgitation rates.

TR grade	Preoperative		First postoperative reports		Most recent postoperative reports	
	No.	%	No.	%	No.	%
None	0	0	5	12.8	6	15.4
Mild	3	7.0	22	56.4	18	46.2
Mild-moderate	25	58.1	9	23.1	11	28.2
Moderate-severe	15	34.9	3	7.7	4	10.3

Prevalence of patients in each grade of tricuspid regurgitations at three different time points: preoperatively, at the time of the first postoperative echocardiogram and at the time of the most recent postoperative echocardiogram. N = 43 preoperatively, n = 39 for both postoperative time points.

## Discussion

Initial results, considering first assessment of regurgitation postoperatively demonstrate this method of repairing the TV was effective. A large majority of patients were free from significant TR. As time progresses, the recurrence and deterioration of TR progresses, with long-term follow-up showing fewer patients free from significant regurgitation; however, there is still significant improvement in grade of regurgitation at this time, when comparing to preoperative status. This progression over time is to be expected, as surgical intervention to treat TR is never a complete cure; this has been established by previous studies looking into durability of TV repair.^8,9^ Reintervention to the TV was not required in any patient. While this points towards efficacy of repair, interpretative caution has to be taken, as tricuspid redo procedures are considered particularly high risk,^10^ with in-hospital mortality rates as high as 37% reported.^11^ As such, redo TV surgery is rarely offered.

Traditionally, De Vega suture annuloplasty was the technique of choice for TV repair; however, in more recent times, there has been increasing evidence that ring annuloplasty has superseded the De Vega technique in most effectively reducing long-term rates of significant TR.^12,13^

One of the main uncertainties that arise with the use of the ring is the type of prosthesis which is preferred. Rigid or semi-rigid rings can be considered over flexible rings, due to evidence supporting a reduced incidence of recurrent TR (10% increase in significant TR with rigid rings compared with a 16% increase in flexible rings over 5 years).^8^ High rates of suture dehiscence have been reported with the use of rigid rings (up to 8.7% in a study from Pfannmüller et al).^14^ The ring approach also poses the potential for compromised ventricular function through fixation of the base of the ventricle. Owing to the nature of using a rigid band, it has been proposed that shearing forces exerted on the septal portion of the annulus are exacerbated when rigid rings are implanted. Alternatively, ‘shallow stitching’ of the ring into the septal annulus could predispose tearing of the ring.^14^ This is less of an issue with suture annuloplasty, given the relatively reduced thickness of a pledget, which can easily be embedded into the annulus. Recently, the development of three-dimensional (3D) rings has been proposed as a solution to ring-related issues, but this is a relatively new area which requires further validation. Cost-effectiveness is a definitive advantage of all suture techniques over ring annuloplasty, as pledgeted sutures are considerably cheaper to source than prosthetic rings.

When comparing to the traditional suture annuloplasty De Vega technique, our approach does offer some benefits. The use of one continuous stitch for De Vega results in a diffuse tightening of the annulus requiring the use of a gauge to approximate and determine the amount of constriction required.^9^ On the contrary, the use of interrupted sutures allows constriction in more specified segments of the annulus in a more step-wise approach, as each stitch is placed. For example, the posterior annulus can first be plicated and then further constrictions applied at each point in which a pledget is placed. Furthermore, if extra competency is required following an on-table water test, additional sutures may be immediately added – this offers a real-time modification to the repair. While modifications of the De Vega annuloplasty with the use of multiple pledgets have been reported, our technique is the first to our knowledge to use multiple interrupted sutures. Durability of De Vega repair has been reported as a rate of recurrent significant TR of around 25%, during follow-up, in some studies.^8^

Similar to dehiscence reported with ring annuloplasty, tissue tearing of sutures in De Vega annuloplasty has been reported as a late complication,^15^ as individual bites in a traditional De Vega annuloplasty are not pledgeted, increasing the risk of tearing. As our technique involves multiple pledgets, there is a theoretical reduced risk of tissue tearing. Indeed, in our follow-up, we found no reports of suture-related tissue damage following annuloplasty. It should be noted that careful suture placement is paramount, as there is a risk of right coronary artery injury if sutures are placed too deeply across the anterior and posterior leaflet aspects of the annuloplasty.

Pathophysiological determinants play an important role both in the initial aetiology of TR and could help to explain recurrence of regurgitation following repair. The main influencers are as follows: left-sided heart disease (LV dysfunction and mitral regurgitation), RV dysfunction and pulmonary hypertension.

**Figure 3.**
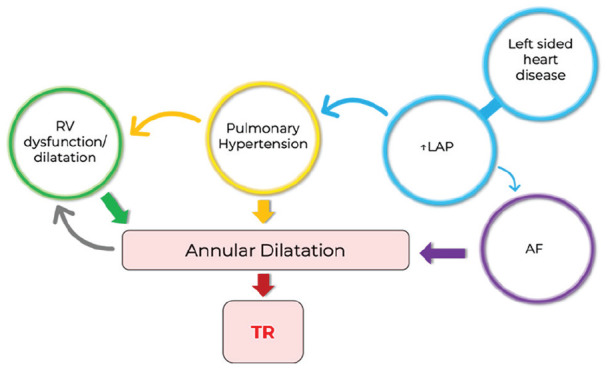
The interplay of pathophysiological determinants on annular dilatation and ultimately tricuspid regurgitation. AF: atrial fibrillation; LAP: left atrial pressure; RV: right ventricle; TR: tricuspid regurgitation.

Tricuspid annular dilatation is the ultimate cause of functional regurgitation which is precipitated by these predictors (Figure 3), with the degree of annular dilatation shown to be directly related to the severity of TR.^16^ A common cause of dilatation is pulmonary hypertension.^17^ Elevated pulmonary artery pressures can create a pressure overload onto the RV, which subsequently leads to RV dilatation and dysfunction.^8^ Right ventricular volume status and function is intimately associated with annular movements. As such, an overloaded and dysfunctional RV can cause annular dilatation resulting in regurgitation.^18^ A functional cycle can then ensue; TR itself can cause further RV dysfunction and dilatation, which worsens annular dilatation and regurgitation.^8^

Meanwhile, left-sided heart disease, such as mitral regurgitation or LV dysfunction, can cause elevated left atrial pressures, which then transmit backwards to the lungs also causing pulmonary hypertension. Another important risk factor for TR is AF. Elevated left atrial pressures and increased atrial size can cause AF, and it then leads to right atrial dilatation, which is also a contributor to tricuspid annular dilatation.^8,19,20^ This could help to explain recurrent rates of TR within our cohort of patients, as the majority of patients had AF.

While our results show a good durability of repair for a suture annuloplasty technique, it is important to elucidate why significant TR recurs. It could either be due to gradual failure of the repair, or due to continued progression of annular dilatation as a result of the pathological determinants aforementioned. In reality, it is likely due to an interplay between the two.

Given the cycle of TR causing RV volume overloading and dysfunction, progressing to annular dilatation and worsening TR, an annuloplasty failure in keeping the annulus tightened and constricted would naturally lead to the recurrence of regurgitation. Furthermore, as annular dilatation is heavily influenced by severity of TR,^16^ the worse the grade of postoperative TR, the greater the dilatory pressures this will put on the repair, thus decreasing the annuloplasty durability.

Our results show a reasonable prospective for the use of interrupted pledgeted suture annuloplasty for TV repair, both in terms of operative safety/efficiency and in durability. However, it cannot be overlooked that our outcomes are mostly initial and midterm. The small sample size of patient’s further limits extrapolation of our findings to a wider population. Furthermore, there was inconsistent echocardiogram reporting both in terms of number of parameters that could be assessed for durability of repair and in terms of time of follow-up. While this is hard to control for, given the number of different echocardiographers and clinicians writing the reports, having a larger sample size would have limited the influence of these incongruencies. Outcomes such as New York Heart Association classification for heart failure were also scarcely reported. This inclusion would have contributed to a morbidity assessment of patients postoperatively. With that said, it would be difficult to tell the sole influence of TV repair on morbidity, given almost all patients had concomitant cardiac procedures with a range of comorbidities likely influencing morbidity status.

## Conclusion

It must be emphasized that our results do not serve to challenge the mostly robust outcomes of larger studies evaluating the use of the current technique of choice, ring annuloplasty. Rather, we report our experience of a novel approach to TV surgery, hoping to contribute to a field of cardiac surgery which is comparatively underreported. These results help to contribute to the wider clinical impression that the TV should not be hesitantly operated upon in the fears of repair failure or increased mortality. Future work should primarily focus on obtaining results from a larger sample of patients with a longer follow-up. Continued follow-up of TV repair patients is currently undergoing in our institution. Comparative analysis between our technique and other forms of suture and ring annuloplasty would also provide value. These comparator studies should form the basis of future research.
